# Are care plans suitable for the management of multiple conditions?

**DOI:** 10.15256/joc.2016.6.79

**Published:** 2016-10-26

**Authors:** Charlotte E. Young, Frances M. Boyle, Allyson J. Mutch

**Affiliations:** ^1^School of Public Health, Faculty of Medicine and Biomedical Sciences, The University of Queensland, Brisbane, QLD, Australia

**Keywords:** Comorbidity, consumer participation, multimorbidity, patient care planning, patient preference, primary care

## Abstract

**Background:**

Care plans have been part of the primary care landscape in Australia for almost two decades. With an increasing number of patients presenting with multiple chronic conditions, it is timely to consider whether care plans meet the needs of patients and clinicians.

**Objectives:**

To review and benchmark existing care plan templates that include recommendations for comorbid conditions, against four key criteria: (i) patient preferences, (ii) setting priorities, (iii) identifying conflicts and synergies between conditions, and (iv) setting dates for reviewing the care plan.

**Design:**

Document analysis of Australian care plan templates published from 2006 to 2014 that incorporated recommendations for managing comorbid conditions in primary care.

**Results:**

Sixteen templates were reviewed. All of the care plan templates addressed patient preference, but this was not done comprehensively. Only three templates included setting priorities. None assisted in identifying conflicts and synergies between conditions. Fifteen templates included setting a date for reviewing the care plan.

**Conclusions:**

Care plans are a well-used tool in primary care practice, but their current format perpetuates a single-disease approach to care, which works contrary to their intended purpose. Restructuring care plans to incorporate shared decision-making and attention to patient preferences may assist in shifting the focus back to the patient and their care needs.

## Introduction

Care plans, widely advocated as a mechanism to individualize chronic disease care [1], are intended to engage primary care clinicians and patients in an examination of clinical evidence and a consideration of patients’ preferences, needs, and values to inform and facilitate care planning and shared decision-making [1, 2]. Empirical evidence indicates that care plans can enhance self-management practices [3], increase adherence to guideline recommendations [4], improve processes and clinical outcomes [5], and reduce or delay hospitalization [6]. However, the effectiveness of care plans relies on the care-planning processes used and the clinician’s and patient’s desire and ability to participate in these processes [7–9].

## Care plans in Australia

Care plans have been used across Canada, Germany, the UK, the USA, and Australia [1]. In Australia, the introduction of the Enhanced Primary Care (EPC) package in 1999 signalled a shift to care planning and a significant change in approach to chronic disease management (CDM) [4, 10]. For the first time, primary care clinicians could be reimbursed by Medicare, Australia’s universal health insurance scheme, for time spent developing multidisciplinary care plans for patients with chronic and complex care needs [4, 10]. In 2005, the EPC was expanded and renamed the Chronic Disease Management items, but care plans remained central to the policy [4, 10].

**Box 1. Medicare requirements for General Practice Management Plans and Team Care Arrangements [11].**

**A General Practice Management Plan must describe:**

- The patient’s healthcare needs, health problems, and relevant conditions
- Management goals with which the patient agrees
- Actions to be taken by the patient
- Treatment and services the patient is likely to need
- Arrangements for providing this treatment and these services
- Arrangements to review the plan by a date specified in the plan

**Team Care Arrangements must describe:**

- Treatment and service goals for the patient
- Treatment and services that collaborating providers will provide to the patient
- Actions to be taken by the patient
- Arrangements to review by a specified date

Care plan templates designed to meet the minimum requirements set by Medicare (see Box 1) and to assist with the development of General Practice Management Plans (GPMPs) (i.e. care plans involving general practitioners [GPs]) and Team Care Arrangements (TCAs) (i.e. care plans led by the GP with involvement from a multidisciplinary team of health professionals) have been developed by governments, and by non-profit and professional organizations [12]. The use of templates is not mandatory, as clinicians can develop their own plan format, but Bolger-Harris *et al*. [12] found that most clinicians prefer to use templates because they are quick, adaptable, increase the chance of reimbursement by Medicare, and provide prompts and checklists for care.

Despite this preference for care plan templates, GPs are critical of their ability to “cater for patients with multiple chronic diseases” [12]. Adding weight to these concerns, the Royal Australian College of General Practitioners (RACGP) [13] questions the ability of care plans to meet the needs of patients and GPs managing multiple chronic conditions. The RACGP argues that the predominant focus on the provision of single-disease care is the most serious gap in Australia’s primary healthcare system, and suggests that the CDM items only add to this issue, as the “needs of complex patients with advanced disease or multiple diseases are not acknowledged” [13].

## Care plans in a multimorbidity context

Empirical evidence informing the development and application of care planning for patients with multiple conditions is needed [1]. A recent Cochrane review of 15 randomized trials examining personalized care planning for adults with chronic conditions found no study that explicitly examined whether care plans led to improved physical, psychological, or subjective health, or improved capabilities of self-management for patients with multiple conditions [1].

Acknowledging the limited evidence informing multimorbidity care [14, 15], researchers have increasingly argued in favour of approaches that move beyond a focus on disease [16–20]. These arguments are grounded within a patient-centred approach, but also stem from empirical work identifying the impractical and potentially hazardous outcomes that can arise when disease-centric interventions that target single conditions (such as clinical practice guidelines) are applied across multiple conditions [14, 21]. Care plans have been emphasized as a mechanism for enhancing the provision of multimorbidity care by supporting patient-centred care [1, 14, 16, 17, 20, 22], although in practice, patients report diverse experiences and different levels of engagement in care-planning processes [9, 23–25]. To achieve greater consistency in care planning and the resultant care plan, broader system-level interventions are required [26, 27]. 

A recent study by Morgan *et al*. [26] trialling care planning for patients with depression, diabetes, and/or coronary heart disease, demonstrates the potential utility of care plans in a multimorbidity context. The trial involved significant investment, with multiple system-level changes, including merging evidence-based guidelines, training primary care practice staff in goal setting and problem solving, changes to practice-based information technology, and automating recall for review [26, 27]. A multiple condition care plan template, informed by clinical practice guidelines, was developed to support clinicians and patients to record and track changes across clinical data for all their conditions. The template required (i) extensive review of the patient’s goals and preferences along with barriers to achievement, (ii) the development of multidisciplinary care arrangements, and (iii) established prompts for guideline-recommended checks [26]. Compared with usual care, patients achieved significant clinical improvements in depression and cardiovascular disease risk [26]. Guideline-recommended checks were also more frequently performed, multidisciplinary care arrangements and communications were well structured and managed, and patients’ goals were comprehensively monitored [26]. Morgan *et al*. [26] concluded that many factors contributed to the success of the trial, but the identification of patients’ goals and priorities, and establishing systems to ensure regular review appeared central [26]. These findings provide preliminary evidence for a patient-centred approach that draws on care planning to enhance multimorbidity care [16, 17, 20, 28], while also highlighting the extent of system-level investment required to support such an approach [26]. 

System-level investment to support the management of multiple conditions is clearly essential, but at a time of fiscal constraint, this is a significant challenge. Major health system reform is difficult and costly to achieve, with most reform arising from incremental change to existing practice [10, 29]. Examining whether current tools that support practice, such as care plan templates, are fit for purpose in the context of multimorbidity care is timely.

## Examining the potential of care plan templates

An examination of care plans and their potential utility for multimorbidity care requires careful consideration of key criteria integral to patient-centred care and shared decision-making. Identifying an empirical evidence base informing appropriate assessment criteria is an obvious challenge, but several leading agencies and authors in the field, such as the American Geriatric Society Expert Panel (AGS) [17], Muth *et al*. [20], and others [28, 30, 31], have proposed key principles to guide the management of multiple conditions within primary care. These principles – developed through extensive literature reviews and in consultation with expert stakeholders [17, 20] – emphasize the need for establishing patient preferences, setting priorities, identifying conflicts and synergies, and establishing review processes. In the absence of a solid evidence base, these components provide a preliminary basis for evaluating whether care plans support the management of multiple chronic conditions.

### Patient preferences

Understanding the health issues, treatments, and agreed actions of significance to the patient, that is, his or her “*preferences*” [18], acknowledges there is rarely a single correct treatment option when managing multiple conditions [32]. Moreover, it recognizes that in managing multiple conditions, a patient’s focus often shifts from disease-specific goals to more global cross-disease outcomes, such as maintenance of physical function, symptom relief, and quality of life [30]. Emphasis on shared decision-making and establishing patient preferences stems from the need to manage the misalignment that can arise between the preferences and goals of patients and those of their clinician [17, 18, 20, 33, 34]. This misalignment may lead patients to disengage from clinical advice, thereby undermining shared decision-making processes [33]. Despite the increasing emphasis on shared decision-making, and taking greater account of what patients want and value [35, 36], its benefits have not been extensively examined [37]. 

### Setting priorities or goals

Overly complex management regimens, conflicts between medications and conditions, and excessive treatment burden are key challenges arising from the management of multiple conditions [22, 38, 39]. Patients overwhelmed by the burden of treatment may not adhere to prescribed treatments [31]. In response, the AGS [17] and Muth *et al*. [20] suggest a patient-centred approach acknowledges that *priorities or goals must be set* in line with the patient’s preferences [17, 20]. In doing so, recognition must also be given as to whether patients wish to participate in goal- or priority-setting decisions [40, 41].

The challenges associated with setting priorities are well documented; with clinicians frequently citing limited resources and the narrow evidence base informing the management of multiple conditions as central to this problem [30, 39]. Clinical practice guidelines, the main drivers of evidence-based care in primary practice, do not account for multimorbidity. Therefore, the information needed to inform goal-setting discussions, such as numbers needed to treat and harm, is often absent or conflicting for patients with multiple conditions [14, 16, 17, 28]. Despite this, evidence suggests that patients with multiple conditions can still engage in shared priority or goal-setting discussions with their clinician, by ranking which broad cross-disease goals are most important to them [30]. Identifying the goals of most importance to the patient is a first step to directing guideline-based, disease-specific care [17, 20, 28]. 

### Conflicts and synergies

The identification of conflicts and synergies is a central part of care planning for patients with multiple conditions, designed to help patients accommodate and avoid being overwhelmed by new conditions [33]. When managing multiple chronic conditions, clinicians often adopt an “additive-sequential model”, in which they examine conditions individually with the most pressing addressed before the consultation ends, and the remainder held over until the next consultation [22]. This process, perpetuated by current Medicare funding arrangements and clinical practice guidelines, reinforces the centrality of individual diseases rather than consideration of conflicts or synergies between them. This can undermine the clinical management of multiple conditions, as it may fail to support patients who place greater importance on function than disease [42]. Opportunities to take a more personalized and holistic view of patient care [43] and to reduce patient burden through processes such as de-prescribing, may also be lost [44]. 

### Regular review

The AGS [17] and Muth *et al*. [20] highlight the need for constant review of patients’ goals, priorities and preferences. This is in keeping with the view that care plans should be living, dynamic documents that change over time and at pivotal points (such as at the time of diagnosis of a new condition [33]), to reflect and support the needs of patients [1, 36]. Regular review of patients’ goals, priorities and preferences ensures care continues to be targeted at the issues of importance and relevance to patients [26]. Review also serves as a means to monitor goals and ensure that patients are supported to work through any barriers that undermine progress [26]. Setting a date for reviewing the care plan is a Medicare requirement (see Box 1), but the scheduled fee for reviewing a GPMP is significantly lower (AUD 72.05) than that for preparing one (AUD 144.25) [45].

In summary, recent evidence [26] suggests that care plans may have the potential to move beyond the management of single conditions and support the provision of multimorbidity care, but this process must be underpinned by key criteria integral to patient-centred care and shared decision-making. The purpose of this study is to review and benchmark existing care plan templates, which include recommendations for comorbid conditions, against the following four criteria: patient preferences, setting priorities, identifying conflicts and synergies between conditions, and setting dates for reviewing the care plan.

## Methods

OvidMedline, Web of science (ISI), Embase, Cinahl, PsycINFO, Cochrane, and PubMed, were searched for care plan templates using the following terms: “patient care planning”, “case management”, “care plan”, and “Australia”. An extensive search of the grey literature was also conducted using Australian websites, including the Department of Health, Department of Veterans’ Affairs, Primary Health Networks, the RACGP, and the websites of relevant non-profit organizations.

The study sought disease-specific (i.e. including pre-filled data related to the specified condition) and generic (i.e. including general headings but no pre-filled information) care plan templates. To be included, a template needed to acknowledge comorbid conditions (e.g. if a care plan template for diabetes also discussed depression, it was included). Templates that acknowledged comorbid conditions were the focus as they were more likely to recognize and support the needs of patients with multiple conditions. Disease-specific templates were also restricted to those that addressed a chronic condition classified as Australia’s National Health Priority areas [46]: cardiovascular health, stroke, cancer (colorectal, lung, breast, and prostate), diabetes, depression, chronic kidney disease, asthma, chronic obstructive pulmonary disease (COPD), arthritis, and musculoskeletal conditions. Additional criteria included care plan templates for the development in primary care practice settings, and applied to individuals aged 18 years and over.

**Figure 1:**
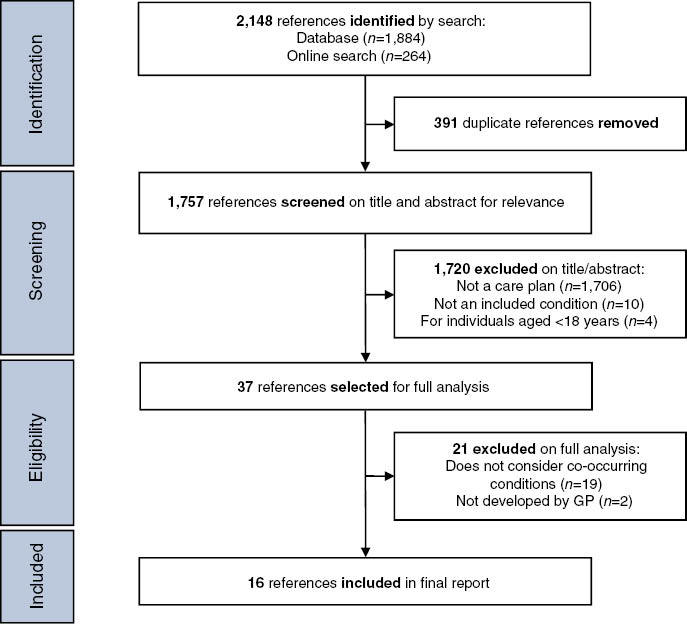
Search strategy care plan. GP, general practitioner.

Figure 1 summarizes the template identification process. After combining the results of all searches and deleting duplicates, 1,757 citations remained. Citations were screened individually based on title and summary; 1,720 were excluded at this point. The full text versions of 37 care plan templates were screened for eligibility. Twenty-one were excluded because they did not consider co-occurring conditions or were not designed for use in primary care.

## Data analysis

Document analysis guided by the framework approach was used to review the care plan templates. The framework approach, which involves five steps (familiarization; identifying a thematic framework; indexing; charting; and mapping and interpretation) [47], was chosen because of its emphasis on applied research that seeks to provide “answers” to clearly established aims [47]. 

To assess the ability of care plan templates to support the management of multiple chronic conditions, the four criteria relevant to care planning for patients with multiple conditions (patient preferences; setting priorities; identification of conflicts and synergies between conditions; and setting dates for reviewing the care plan) were used to construct a data-extraction index or thematic framework (Table 1). The thematic framework was used for coding, with relevant passages from each care plan template extracted in accordance with identified themes and placed in charts to assist with mapping and interpreting the data.

## Results

Sixteen care plan templates [48–63], thirteen designed for specific diseases [48–54, 57, 59–63] and three generic ones [55, 56, 58] developed to cover various conditions met the inclusion criteria. Of the thirteen disease-specific templates, four were for cardiovascular health [48, 53, 59, 61], four for musculoskeletal conditions [50–52, 54], two for diabetes [62, 63], one for depression [49], one for COPD [60], and one for mental health [57]. Seven care plan templates were GPMPs [49, 51, 52, 55, 57, 59, 63], four were TCAs [50, 54, 56, 61], four were combined GPMPs and TCAs [48, 53, 60, 62], and one was for the Coordinated Veterans’ Care (CVC) Program [58]. The CVC Program is an initiative of the Department of Veterans’ Affairs, which provides reimbursement to primary care clinicians who develop care plans for veterans and eligible relatives with one or more chronic conditions or complex care needs [64]. Ten care plan templates were developed by Primary Health Networks (formerly Medicare Locals) [48–54, 60–62], three by the Department of Health [55–57], one by the Department for Veterans’ Affairs [58], one by the RACGP [63], and one by the Heart Foundation [59].

All of the disease-specific templates [48–54, 59–63], with the exception of the GPMP template for mental health [57], included some pre-filled information (see Table 2A). This information, drawn from clinical practice guidelines, was provided under each of the care plan headings or components for the specific condition. For example, the GPMP template for depression [49] contained pre-filled information on the patient’s problems, goals, and required treatments and services, including patient actions, and arrangements for treatments/services for depression. Pre-filled data on the patient’s health issues, corresponding goals, treatments, and agreed actions, were organized under similarly labelled subheadings across the disease-specific templates: general, lifestyle, biomedical, medication, and psychosocial. The generic templates [55, 56, 58] did not include pre-filled information; instead, they provided the headings (e.g. health issues, goal, required treatments, and agreed actions) under which primary care clinicians and patients can record information (see Table 2B). The GPMP template for mental health was the only disease-specific template that did not include pre-filled data [57]. This template includes many of the same headings included in the generic templates (i.e. patient needs, goals, treatments), and headings related specifically to mental health (i.e. results of mental state examination, crisis/relapse) [57].

Data relating to the four criteria relevant to care planning for patients with multiple conditions are outlined below. Table 3 summarizes the extent to which each of the assessed templates addressed the four criteria.

## Patient preferences

Across the templates, the broad criterion of patient preferences was mapped against three subthemes: a description of the health issue(s); management goals; and treatments and agreed actions. Eleven care plans [48, 49, 51–53, 55, 57, 58, 60, 62, 63] required the patient’s health issues or conditions be recorded. For example, the GPMP template for diabetes included the following heading, “patient’s problems/needs/relevant conditions”, under which clinicians and patients could respond [63]. Two templates [48, 60], both combined TCAs/GPMPs, extended this request for information by encouraging primary care clinicians and patients to independently record the health issues. Of the five templates that did not request information on the health issue(s) [50, 54, 56, 59, 61], four were TCA templates [50, 54, 56, 61], which are not required by Medicare, to record patients’ health issue(s). 

Fifteen care plan templates [48–55, 57–63] provided a heading under which goals for care could be recorded; for example, the TCA template for osteoarthritis flagged “goals to be achieved” [54]. Six templates [53, 55, 57–59, 62] stipulated patients should agree to the goals for care; for example, the generic GPMP template included the heading “management goals with which the patient agrees” [55]. The generic TCA template [56] did not record patients’ management goals, but rather, focused on treatment and service goals.

All of the care plan templates recorded the treatments and/or agreed actions [48–63]. For example, the GPMP for depression flagged “treatment and services required, including actions to be taken by the patient” [49]. Four templates [48, 58–60] stipulated that the primary care clinician should agree to these treatments or actions by the patients, as demonstrated by the heading “agreed action by health professionals and patients” in the GPMP/TCA template for COPD [60].

## Setting priorities

The priority-setting theme included processes to assist clinicians and patients to prioritize patient preferences. Only three of the care plan templates included priority-setting processes [53, 58, 62]. Two of these templates, the GPMP template for diabetes and the GPMP/TCA template for hypertension, addressed this criterion in a rudimentary way by simply recording the “primary diagnosis/main issue” [53, 62]. The third template, for the CVC Program, asked the patient to identify and rate their problems (on an eight-point scale: from 0=“not at all”, to 8=“a lot”) [58]. The template also asked patients to identify a goal and to rate their progress in achieving that goal (on an eight-point scale: from 0=“no success”, to 8=“complete success”) [58]. Notably, the template only included space for one problem statement and one goal statement, but additional statements could be added elsewhere [58]. 

## Identifying conflicts and synergies between conditions

Processes for identifying conflicts and synergies between conditions were not flagged by any of the care plan templates. 

## Setting dates for reviewing the care plan

In line with the minimum requirements set by Medicare, all of the care plan templates [48–61, 63], with the exception of the GPMP/TCA template for diabetes [62], requested a review date for the care plan, but only two templates requested a review of the patient’s management goals.

## Discussion

Sixteen care plan templates were identified to assess their ability to support shared decision-making and enhance the management of multiple conditions. None of the care plan templates addressed all of the criteria (patient preferences; priority settings; identification of conflicts and synergies between conditions; and setting dates for review of the care plan), but most addressed one or more to some extent. Patient preference, linked to three subthemes (health issue(s); management goals; and treatments and agreed actions) was the most commonly addressed criterion, while substantially less emphasis was placed on priority settings and the review of individual management goals. None of the care plan templates identified conflicts and synergies. 

Thirteen of the reviewed care plans were pre-filled disease-specific templates. The inclusion of pre-filled data in care plan templates, while important for bringing evidence-based medicine to the point of practice, runs the risk of overshadowing genuine care-planning discussions. Care planning is intended to involve both primary care clinicians and patients reflecting on clinical evidence and patient preferences, to inform and facilitate shared decision-making, resulting in the development of a joint care plan for managing the patient’s condition(s) [1]. In contrast, the pre-filling of templates can direct care-planning discussions to focus on the issues, goals, treatments, and to agree on actions recommended by disease-specific clinical practice guidelines. In essence, the discussion remains one sided with the emphasis being placed on clinical decision-making and “medical agendas” [25], rather than supporting genuine care planning discussions and consideration of patient preferences for care. This is particularly concerning for patients with multiple conditions who may often have contraindications to the treatments recommended in disease-specific guidelines [14].

In line with the single-disease approach, for which clinical practice guidelines are often criticized [14, 21], the care plan templates guided clinicians to consider co-occurring health issues in a sequential manner. Isolating the management of conditions in this way may not reflect the way patients think or prioritize care [22, 30, 33]. When considering trade-offs between competing conditions, medications and treatments, some patients often shift their focus from disease-specific outcomes to more global health outcomes, such as maintenance of physical function, symptom relief, and quality of life [15, 30], while others prefer not to acknowledge individual conditions [41]. Fried *et al*. [30] suggest having patients identify or prioritize the global health outcomes of importance to them and to organize care planning around those outcomes. Pre-filled information sees the direction and focus of care at least partially pre-determined, while “data field” requirements under current GP Medicare funding arrangements may present a further barrier to shared decision-making between clinicians and patients. Swinglehurst *et al*. [24] and Blakeman *et al*. [25] also made similar observations when examining the use of care plan templates in UK primary care practices. Disease-specific care plan templates directed care-planning discussions towards “medical agendas”, while completing specified “data fields” to meet set quality indicators impeded the clinician’s ability to engage in genuine care-planning processes. These findings, in combination with our own, suggest that care plans have drifted from their intended purpose of fostering patient-centred care to driving clinicians to meet policy requirements. Pre-populated care plans have a place in current primary care practice as indicated by the expressed preferences of clinicians [12]. However, our findings support calls for pre-populated disease-specific information to serve as a means of achieving the broader health goals identified by the patient, and not as a goal in itself [20, 28, 30]. 

Few of the care plan templates reinforced the need to engage patients in the development of care plans or included processes to assist clinicians and patients to set priorities. It is also important to note that some patients may not wish to participate in care decisions, but this should be an informed rather than an imposed choice [65]. When faced with managing numerous potentially conflicting conditions, often with limited time and resources, some patients and clinicians will set priorities, but these can differ [33, 34]. Working through differing priorities can assist to increase patients’ adherence with prescribed care [31], reduce the complexity or treatment burden faced by patients [31], and ensure that the care plan addresses the issues of importance to patients [34]. Yet seeking “agreement” with patients was not comprehensively encouraged or supported by the templates. Similarly, templates did not seek to reduce the complexity of care management through the identification of conflicts and synergies. Research suggests that primary care clinicians rarely initiate priority-setting discussions with their patients [66]; it is unclear whether this is due to limited availability of priority-setting tools [38, 39, 67] or to the culture of current practice [67]. Clinicians have called for methods to support shared decision-making and to resolve potential differences between their priorities and those of their patients with multiple conditions [38, 39]. By not encouraging priority setting and regular review of patients’ priorities and goals for care, care plans are missing a valuable opportunity to assist both clinicians and patients to manage multiple conditions. 

Overall, our findings suggest that current care plan templates may inadvertently impede, rather than foster, shared decision-making, but there is scope for care plans to support the management of multiple conditions. This was demonstrated by the generic CVC Program template, which addressed the majority of the criteria, and included comprehensive methods for setting priorities. The generic CVC Program template encouraged patient ownership of the plan, using headings such as “Identified issues (including self-management)”, “What I want to achieve?” and “Steps to get there”. The template also encouraged the review of individual goals and the documentation of patient progress towards that goal, allowing for potential barriers or enablers to care to be identified. The CVC Program template offers a clear example of how templates might be improved to better facilitate shared decision-making and multimorbidity care. Care plans are but one component of the wider system in which multimorbidity care takes place. To make meaningful and sustainable change, modifications to improve their relevance in a multimorbidity context, must be underpinned by, broader system level interventions. Some of these system level changes are already underway. The recent Primary Health Care Advisory Group (PHCAG) report recommends changes to Australia’s current health management and funding models, placing greater emphasis on patient-centred care and shared decision-making in primary care settings [36]. Care plans are explicitly identified as a means of facilitating patient-centred care and shared decision-making with providers encouraged to: “*Work with and support their patients to set shared goals and make shared decisions about the inclusions of their care plan that are aligned and appropriate to their needs, circumstances, preference and context*” [36]. 

The report also cautions against the use of “expressly automated” care plans, targeted more “towards satisfying requirements for payment rather than the needs of the patient” [36]*.* In the May 2016 federal budget, the Australian Government announced a AUD 21 million commitment to trial the recommended changes outlined in the report [68]. 

The limitations of this study need to be acknowledged. An extensive search was conducted, but it is possible that eligible Australian care plan templates were missed. No formal or tested criteria currently exist for evaluating care plan templates in a multimorbidity context. The evaluation criteria used in this study were drawn from current evidence, but it is possible that relevant questions or criteria were not considered. In addition, due to the limited evidence base for multimorbidity interventions and management [1, 15], we do not know if adopting care plan templates based on the domains suggested will impact patient outcomes. Nonetheless, the findings highlight a number of issues that are potentially important in shaping the management of multiple conditions. This study evaluated care plan templates and not the manner in which they are used by clinicians or patients. It is possible that clinicians engage in priority-setting discussions and the identification of conflicts and synergies without these being flagged in current templates. However, current research suggests this is not generally the case. This study focused on care plan templates developed for use in Australian primary care practice and as a result, the care plans assessed were structured towards meeting the requirements set by Medicare and Australian clinical practice guideline recommendations. However, our findings are consistent with those reported by Swinglehurst *et al*. [24] and Blakeman *et al*. [25], suggesting they may have broader implications for the design of care plan templates beyond the Australian context. 

## Conclusion

Care plans are a well-used tool in primary care practice, but their current format perpetuates a single-disease approach to care, which works contrary to their intended purpose. Policy constraints, medical agendas, and clinical practice guidelines strongly influence the use of care plans in current practice. Restructuring care plans to incorporate shared decision-making and attention to patient preferences may assist in shifting the focus back to the patient and their care needs.

## Figures and Tables

**Table 1:** Care plan framework themes.

Theme	Description
Patient preferences	Health issues identified by patients, patient preferences for management goals, process or treatment choice
Setting priorities	Processes to assist clinicians and patients to prioritize health issues/conditions, management goals, outcomes, treatments, or services
Identifying conflicts and synergies between conditions	Identification of conflicts between conditions, medications, and management strategies; barriers to following the care plan; commonalities between conditions, medications, and management strategies; and enablers to following the care plan
Setting dates for reviewing the care plan	Setting dates for reviewing patient goals and priorities

**Table 2A:** Extract from a disease-specific care plan template [49].

GP MANAGEMENT PLAN – MBS ITEM NO. 721 (DEPRESSION/ANXIETY DISORDER)			
Patient problems/needs/relevant conditions	Goals – changes to be achieved (if possible)	Required treatments and services, including patient actions	Arrangements for treatments/services (when, who, and contact details)
1. *General*			
Patient’s understanding of depression/anxiety	Patient to increase their understanding of depression/anxiety and how it can be managed	Patient education	GP
			Nurse
			Allied health professional
Symptoms	Improve mood, sleep, energy, attention, concentration, motivation, sexual function	Therapy consider:	GP
	Improve physical symptoms (e.g. fatigue, headache, muscle pains, weight loss)	– CBT	Allied health professional
	Increase self confidence	– Counselling	Psychiatrist
	Identify and address thoughts related to suicide	– Psychotherapy	
		– Relaxation training	
Causes/stressors and precipitants	Identify stressors and precipitants, such as relationship and family problems, negative thinking, loss and grief, coexisting physical conditions	Counselling consider:	GP
		– Problem solving	Patient
		– CBT	Allied health professional
		– Interpersonal therapy	Psychiatrist
		– Marital/family therapy	
		– Loss/grief counselling	
Maintenance/relapse prevention	Avoid relapse/decrease severity of relapse	Regular review	GP
	Increase awareness of stressors/circumstances that could trigger a relapse	Address stressors and known risk factors for relapse	Patient
		Early intervention of a recognized relapse	Allied health professional
			Psychiatrist

CBT, cognitive behavioural therapy; GP, general practitioner; MBS, Medicare Benefits Schedule.

**Table 2B:** Extract from a generic care plan template [55].

PREPARATION OF A GP MANAGEMENT PLAN (ITEM 721)			
Patient’s health problems/health needs/relevant conditions	Management goals with which the patient agrees	Treatment and services required, including actions to be taken by the patient	Arrangements for providing treatment/services (when, who, contact details)




**Copy of GPMP offered to patient?** YES/NO			
**Copy/relevant parts of the GPMP supplied to other providers?** YES/NO/NOT REQUIRED			
**GPMP added to the patient’s records?** YES/NO			
**Review date for this plan:** dd/mm/yy			
The referral form issued by the Department can be found at www.health.gov.au/mbsprimarycareitems or a form can be used that contains all of the components of the Department’s form.			

GP, general practitioner; GPMP, General Practice Management Plan.

**Table 3:** Appraisal of the included care plans according to criteria.

Care plan template [reference]	Patient preferences			Priority settings	Identification of conflicts and synergies between conditions	Review*
	Patient identified health issues	Patient identified management goals	Patient agreed treatments and actions			
GPMP generic [42]	+	++	+	–	–	+
GPMP diabetes [50]	+	+	+	–	–	+
GPMP CHD [46]	–	++	++	–	–	+
GPMP depression [36]	+	+	+	–	–	+
GPMP osteoarthritis [38]	+	+	+	–	–	+
GPMP osteoporosis [39]	+	+	+	–	–	+
GPMP mental health; generic [44]	+	++	+	–	–	+
TCA generic [43]	–	–	+	–	–	+
TCA CHD [48]	–	+	+	–	–	+
TCA osteoporosis [37]	–	+	+	–	–	+
TCA osteoarthritis [41]	–	+	+	–	–	+
CVC Program generic [45]	+	++	++	++	–	++
GPMP and TCA diabetes [49]	+	+	+	+	–	–
GPMP and TCA COPD [47]	++	+	++	–	–	+
GPMP and TCA cardiac [35]	++	+	++	–	–	++
GPMP and TCA hypertension [40]	+	++	+	+	-	+

*Setting dates for reviewing the care plan; –, does not address criteria; +, somewhat addresses criteria; ++, addresses criteria.

CHD, coronary heart disease; COPD, chronic obstructive pulmonary disease; CVC, Coordinated Veterans’ Care Program; GPMP, General Practice Management Plan; TCA, Team Care Arrangement.
